# Serological and virological surveillance of avian influenza virus in domestic ducks of the north-east region of Bangladesh

**DOI:** 10.1186/s12917-017-1104-6

**Published:** 2017-06-17

**Authors:** Rahul Deb Sarker, Mohammad Giasuddin, Emdadul Haque Chowdhury, Mohammad Rafiqul Islam

**Affiliations:** 1 Department of Pathology, Bangladesh Agricultural University, Mymensingh, Bangladesh; 2 National Reference Laboratory for Avian Influenza, Bangladesh Livestock Research Institute, Savar, Dhaka Bangladesh

**Keywords:** Avian influenza virus, Ducks, Seroprevalence, Molecular characterization, Phylogenetic analysis

## Abstract

**Background:**

Wild waterfowl are considered as the natural reservoir for avian influenza (AI) viruses. Bangladesh has been experiencing highly pathogenic avian influenza (HPAI) outbreaks since 2007, mostly in chickens and occasionally in ducks. Ducks play an important role in the persistence and genetic recombination of AI viruses. This paper presents the results of serological and virological monitoring of AI in domestic ducks in 2013 in the north-east region of Bangladesh.

**Results:**

A total of 871 and 662 serum samples and 909 and 302 pairs of cloacal and oropharyngeal swabs from domestic ducks of Mymensingh and Sylhet division, respectively, were analysed. Antibodies to type A influenza virus were detected by blocking ELISA in 60.73 and 47.73% serum samples of Mymensingh and Sylhet division, respectively. On haemagglutination-inhibition (HI) test 17.5% of ELISA positive serum samples were found to be seropositive to H5 avian influenza virus. Five cloacal swabs and one oropharyngeal swab were positive for M gene of type A influenza virus by real time RT-PCR (rRT-PCR), but all of them were negative for H5 influenza virus. Three of the six viruses were successfully characterized as H1N5, H2N5 and H7N5 subtype of AI virus, the other three remained uncharacterized. On sequencing and phylogenetic analysis the HA and NA genes were found to be of Eurasian avian lineage. The H7 virus had cleavage site motif of low pathogenic virus.

**Conclusions:**

Low pathogenic avian influenza viruses were detected from apparently healthy domestic ducks. A small proportion of domestic ducks were found seropositive to H5 AI virus.

## Background

Wild waterfowl serve as the natural reservoir for Type A influenza viruses and play an important role in the persistence and transmission of these viruses among birds and other mammalian species. Influenza A viruses are classified into subtypes based on two surface proteins, the hemagglutinin (HA) and neuraminidase (NA). At least 16 hemagglutinins (H1 to H16) and 9 neuraminidases (N1 to N9) have been found in AI viruses from birds [1], while two additional HA and NA types have been identified only in bats [2]. Influenza viruses in birds are classified as either low pathogenic avian influenza (LPAI) or highly pathogenic avian influenza (HPAI) viruses. The HPAI viruses that produce acute clinical disease in chickens, turkeys and other birds belong to either H5 and H7 subtypes. Many different subtypes of AI viruses resulting from the combination of the 16 HA and 9 NA antigens have been found in wild waterfowl [3]. Low pathogenic AI viruses of H5 or H7 subtypes also may persist in wild ducks, which may evolve into HPAI viruses. The ancestor of the currently circulating H5N1 HPAI viruses was first detected in bar-headed goose in Guangdong province, China in 1996. Following some genetic recombinations, the global dissemination of this H5N1 virus began in 2003 with devastating consequences for the poultry industry [4]. In Bangladesh H5N1 HPAI virus was first detected in 2007 [5, 6] and 550 outbreaks have been reported to OIE until now [7]. Most of the outbreaks were in chickens, but clinical disease also has been observed in quails, crows, ducks and geese [8–10]. Three different genetic clades of HPAI H5N1 virus, namely clade 2.2, 2.3.4 and 2.3.2.1 were detected [8, 11, 12]. As of 19 July 2016, 8 human cases of H5N1 AI virus with one case fatality have been reported from Bangladesh [13]. In addition to H5N1 HPAI, low pathogenic H9N2 influenza virus also has been circulating in poultry [14–16].

Total population of domestic ducks (*Anas platyrhynchos*) in Bangladesh is 48.86 million [17]. Ducks are reared either in scavenging or semi-intensive system. In scavenging system chickens and ducks are usually reared in the same household. In semi-intensive system ducks are brought to the rivers and lakes for feeding, where they get a chance to mingle with the wild waterfowl. Thus AI viruses can transmit from wild waterfowl to domestic ducks. The present paper reports on the serosurveillance and molecular detection and characterization of AI viruses from domestic ducks in north-eastern districts of Bangladesh.

## Methods

### Sample collection

**Fig. 1:**
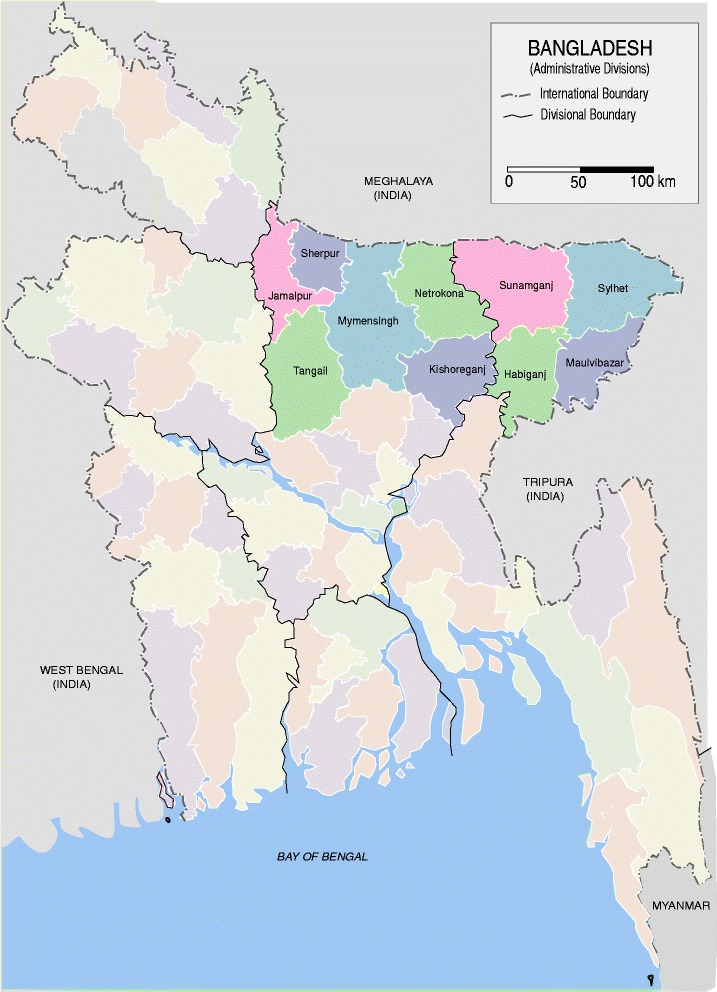
The *map of Bangladesh* showing the areas of sample collection (*shaded and labelled*). (Map template source: https://commons.wikimedia.org/wiki/File:BD_Map_admin.svg)

An AI surveillance programme was conducted by the Department of Livestock Services (DLS), Bangladesh in collaboration with several national laboratories with assistance from the Food and Agriculture Organisation (FAO) of the United Nations in 2013. The samples we used in this study were collected through this surveillance platform. This surveillance program targeted ducks and swine population of the country. Samples collected by DLS veterinarians from different regions of the country were sent to the nearby national laboratories. Our laboratory was entrusted with the responsibility of analyzing samples from the north-western region of Bangladesh including Mymensingh division (Jamalpur, Sherpur, Tangail, Mymensingh, Netrokona and Kishoreganj districs) and Sylhet division (Sunamganj, Hobiganj, Sylhet and Moulvibazar districts) (Fig. 1). Duck rearing is popular in this region because of the presence of many natural waterbodies. With the exception of Netrokona district there was no clinical outbreak of AI in ducks during the period from 2007 to 2013. Serum samples and cloacal and oropharyngeal swabs of 909 and 671 domestic scavenging or semi-scavenging ducks of more than 8 weeks of age from the Mymensingh and Sylhet division, respectively, were collected during the period from January to August 2013. Cloacal and oropharyngeal swabs were placed individually in cryovials containing virus transport medium. The transport medium was prepared with Dulbecco’s modified Eagle Medium supplemented with HEPES (20 mM), L-glutamine (2 mM), gentamycin (250 μg/ml), sulfamethoxazole (200 μg/ml), ofloxacin (60 μg/ml), polymyxin B (2000 IU/ml), amphotericin B (2 μg/ml) and bovine serum albumin (2.5%). The samples were shipped to the laboratory either in a cooler with ice pack or in liquid nitrogen, depending on the time required for transportation. Blood samples were also collected from the wing vein of the ducks. The blood was allowed to clot and the serum was separated by centrifugation using a portable centrifuge, and shipped to the laboratory maintening the cool chain as mentioned above.

### Detection of antibodies to influenza virus

A blocking ELISA kit (IDEXX AI Multi S-Screen ELISA kit, IDEXX Laboratories, USA) was used to measure antibodies to Type A influenza virus in all the duck serum samples according to manufacturer’s instruction. Positive and negative control samples provided with the test kit were included on each plate. Serum samples with a sample-to-negative control (S/N) absorbance ratio less than 0.50 were considered positive.

A total of 80 serum samples out of 845 ELISA-positive samples were randomly selected. The selected samples had a wide range of ELISA values; S/N absorbance ratio 0.10–0.19 (*n* = 27), 0.20–0.29 (*n* = 16), 0.30–0.39 (*n* = 24) and 0.40–0.49 (*n* = 13). These samples were tested for antibodies to H5 avian influenza virus by haemagglutination-inhibition (HI) test. The H5 antigen (inactivated allantoic fluid containing H5N1 avian influenza virus of clade 2.3.2.1a virus) was obtained from the National Reference Laboratory for Avian Influenza, Bangladesh Livestock Research Institute, Savar, Dhaka. The HI test was performed as described in OIE manual [18].

### Molecular detection of avian influenza virus

Viral RNA was extracted from swab samples with KingFisher ML automated RNA extraction machine (Thermo Scientific, USA) using Ambion MagMAX Viral RNA Isolation Kit (Life Technologies, USA). Extraction protocol provided by the kit manufacturer was adapted to KingFisher ML automated RNA extraction machine.

All samples were first screened for the presence of Type A influenza virus by Taqman real time RT-PCR for M gene [19]. The real time RT-PCR was performed on AB 7500 Fast Real Time PCR machine (Applied Biosystems Inc., USA). The reaction was performed in MicroAmp Optical 96 Well Fast PCR plates using AgPath-ID™ One Step RT-PCR kit (Life Technologies, USA) following manufacturer’s instruction. The primer and probe sequences are given in Table 1.

**Table 1:** Oligonucleotide primers and probes used in RT-PCR

Target Gene	Primer/Probe Name	Sequence	Product size(bp)	Reference
M gene (Type A AIV) (TaqMan Real time RT-PCR)	M + 25P F	5′-AGA TGA GTC TTC TAA CCG AGG TCG-3′	100 bp	[20]
	M-124 R	5′-TGC AAA AAC ATC TTC AAG TCT CTG-3′		
	M + 64 Probe	[FAM] 5′-TCA GGC CCC CTC AAA GCC GA-3′ [BHQ1]		
HA gene (H5 AIV)	H5–155 F	5′-ACA CAT GCY CAR GAC ATA CT-3’	545 bp	[21]
	H5-699R	5′-CTY TGR TTY AGT GTT GAT GT-3′		
HA gene, full-length (All Type A AIV)	Bm-HA-1F	5′-TATTCGTCTCAGGGAGCAAAAGCAGGGG-3′	1778 + 29 bp	[22]
	Bm-NS-890 (HA) F	5′-ATA TCG TCT CGT ATT AGT AGA AAC AAG GGT GTT TT-3′		
HA gene, partial (All Type A AIV)	HA-1134F	5′-GGR ATG RTH GAY GGN TGG TAY GG-3′	640 bp	[23]
	Bm-NS-890 (HA) R	5′-ATA TCG TCT CGT ATT AGT AGA AAC AAG GGT GTT TT-3′		[22]
NA gene, full-length (All Type A AIV)	Ba-NA-1F	5′-TAT TGG TCT CAG GGA GCA AAA GCA GGA GT-3’	1413 + 29 bp	[22]
	Ba-NA-1413R	5′-ATATGGTCTCGTATTAGTAGAAACAAGGAGTTTTTT-3′		

All M gene positive samples were then tested for H5 avian influenza virus by conventional RT-PCR for H5 gene [20]. The one step RT-PCR was performed on Eppendorf Mastercycler Gradient thermocycler (Eppendorf, Germany) using SuperScript® III One-Step RT-PCR System with Platinum® Taq DNA polymerase (life Technologies, USA) following manufacturer’s instruction. The primer sequences are given in Table 1.

### Attempted propagation of virus in chicken embryos

Propagation of virus in chicken embryos from M gene positive samples was attempted following the standard procedure of embryo inoculation by the allantoic route [18]. The growth of virus in the allantoic fluid was checked by haemagglutination test and RT-PCR. One more blind passage was performed for negative samples.

### Sequencing of HA and NA gene

The virus that could be grown in embryos was sent to OIE-FAO Reference Laboratory, Padova, Italy for genetic characterization. If any M gene posititive sample could not be propagated in embryos, then the RNA isolated from the original swab sample was used for direct amplification of full-length or partial HA and NA genes by one step RT-PCR using universal primer set [21, 22] and SuperScript® III One-Step RT-PCR System with Platinum® Taq DNA polymerase (life Technologies, USA). Primer sequences are given in Table 1. Amplified cDNA was cleaned by gel extraction method using Wizard® SV Gel and PCR Clean-Up System (Promega, USA) according to manufacturer’s instruction. The purified RT-PCR products were sequenced in a commercial laboratory (1st Base, Malaysia) using PCR primers.

### Phylogenetic analysis

The raw sequence data were first checked for its quality and then edited and assembled using softwares Chromas (http://technelysium.com.au), Lasergene EditSeq and MegAlign (DNASTAR Inc., USA). Edited sequences were subjected to BLAST search in the GenBank to find the sequences closely related to the sequences established in the present study. HA and NA sequences of the same subtype representing different genetic lineages were downloaded from the GenBank and used in phylogenetic analysis. The multiple alignment using Clustal W algorithym and construction of maximum likelihood phylogenetic tree were performed with MEGA 5.1 software (http://www.megasoftware.net).

## Results

### Serosurveillance

A total of 871 and 662 serum samples out of 909 and 671 samples collected from Mymensingh and Sylhet division, respectively, were tested by ELISA for antibodies to type A influenza viruses. The remaining 47 samples were not suitable for testing due to excessive hemolysis. The results are presented in Table 2. The prevalence of seropositive ducks in Mymensingh division ranged from 41.48 to 86.24% during different months and the overall prevalence was 60.73% (Table 2). The prevalence of seropositive ducks was higher during January to April as compared to that during May to July. From Sylhet division the samples were obtained during the period from June to August and the prevalence of seropositive ducks was 40.41 to 52.94%, with overall prevalence of 47.73%. The overall seroprevalence in Mymensingh and Sylhet divisions together was 55.12%.

**Table 2:** Seroprevalence of type A influenza virus in ducks of Mymensingh and Sylhet divisions during January to July 2013

Month	No. of samples tested	No. of samples positive	% of sample positive
*Mymensingh division*			
January 2013	78	61	78.20%
February 2013	174	105	60.34%
March 2013	134	111	82.83%
April 2013	109	94	86.23%
May 2013	112	60	53.57%
June 2013	170	59	34.70%
July 2013	94	39	41.48%
Total	871	529	60.73%
*Sylhet division*			
June 2013	146	59	40.41%
July 2013	125	50	44.00%
August 2013	391	207	52.94%
Total	662	316	47.73%
Overall	1533	845	55.12%

**Fig. 2:**
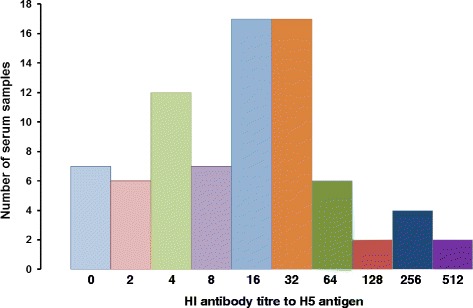
HI antibody titre against H5 antigen among Type A avian influenza ELISA-positive serum samples from domestic ducks

A total of 80 randomly selected ELISA-positive serum samples were tested for antibodies to H5 influenza virus by HI. Out of 80 selected ELISA-positive samples 32 had H5 HI antibody titre less than 16, which is usually considered non-specific [18], and 48 had HI antibody titre between 16 and 512 (Fig. 2). Considering HI titre >32 as H5-specific antibody titre, only 14 out of 80 (17.5%) ELISA positive samples were H5 positive.

### Virological surveillance and molecular characterization of the virus

For virological surveillance all the 909 pairs of oropharyngeal and cloacal swabs from ducks of Mymensingh division and 302 pairs (out of 671 pairs) of oropharyngeal and cloacal swabs from ducks of Sylhet division were tested for M gene of type A influenza virus by rRT-PCR. The remaing 369 pairs of samples from Sylhet division could not be tested due to shortage of reagents. Only six samples (0.5%) including one oropharyngeal swab and 5 cloacal swabs were positive for type A influenza virus; all the positive samples were from Mymensingh division. However, all the 6 M gene positive samples were negative for H5 gene on RT-PCR.

Out of 6 M gene positive samples, only one (22-C) could be successfully propagated in chicken embryos. This sample was charactecterized as H7N5 virus at OIE-FAO Reference Laboratory for Avian Influenza, Padova, Italy. The laboratory has made the HA gene sequence of this isolate publicly available in the GenBank (accession no. KM267817). Examination of the deduced amino acid sequence reveals that this H7N5 virus is a low pathogenic one with cleavage site sequence PELPKGR*GLF. The HA gene sequence of this isolate was downloaded from the GenBank and included in phylogenetic analysis in the present study.

**Fig. 3:**
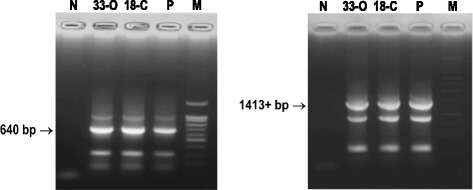
Amplification of HA (left) and NA (*right*) gene of avian influenza virus by RT-PCR for sequencing. (M = marker, P = positive control, N = water control, 330 and 18C are two samples)

For the remaining 5 samples, which were positive for M gene in rRT-PCR but could not be propagated in embryos, molecular characterization was attempted from the RNA extracted from the original swab samples. We were successful in amplifying nearly full-length NA gene (1413+ bp) and partial HA gene (640 bp) from two samples (18-C and 33-O) by RT-PCR (Fig. 3). Nucleotide sequence of the amplified RT-PCR products were established and submitted to GenBank (Accession No. KX185879, KX185880, KX185881, KX185882). Amplification of full-length HA gene was not successful. The amplified 640 bp fragment of the HA gene was from the 3′ end and that did not cover the cleavage site. BLAST search for homology in the GenBank revealed that the virus from the sample 18-C and 33-O belonged to H1N5 and H2N5 subtype of avian influenza virus, respectively. The summary results of molecular characterization of the viruses are presented in Table 3.

**Table 3:** Molecular characterization of avian influenza viruses detected from apparently healthy domestic ducks of Mymensingh division in 2013

Sample ID	Sample type	C_T_ value in rRT-PCR for Type A influenza virus M gene	Propagation in chicken embryos	Subtyping result
18-C	Cloacal swab	26	- ^a^	H1N5
122-C	Cloacal swab	35	-	-
22-C	Cloacal swab	35	Yes	H7N5
23-C	Cloacal swab	35	-	-
25-C	Cloacal swab	35	-	-
33-O	Oropharyngeal swab	34	-	H2N5

^a^Not successful

### Phylogenetic analysis

**Fig. 4:**
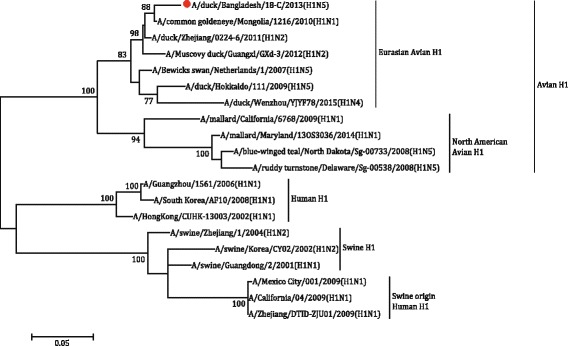
The Maximum Likelihood (ML) phylogenetic tree based on the partial H1 gene sequences (600 bp) of H1N5 virus of the present study and representative H1 viruses of different genetic lineages. The *scale* indicates the number of substitutions per site. The H1N5 sequence of the present study is indicated with a closed *circle symbol*

**Fig. 5:**
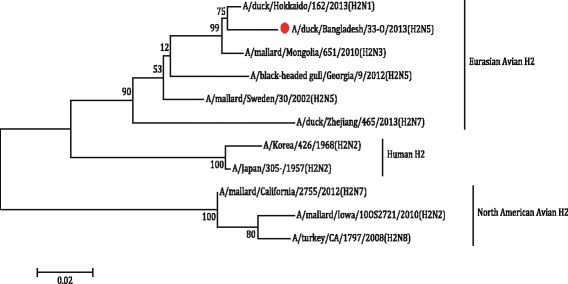
The Maximum Likelihood (ML) phylogenetic tree based on the partial H2 gene sequences (600 bp) of H2N5 virus of the present study and representative H2 viruses of different genetic lineages. The *scale* indicates the number of substitutions per site. The H2N5 sequence of the present study is indicated with a closed *circle symbol*

**Fig. 6:**
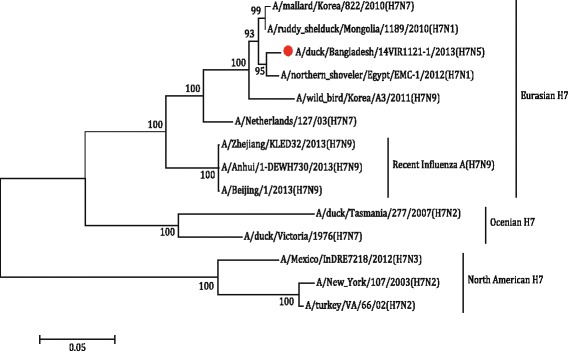
The Maximum Likelihood (ML) phylogenetic tree based on the H7 gene sequences of H7N5 virus of the present study and representative H7 viruses of different genetic lineages. The *scale* indicates the number of substitutions per site. The H7N5 sequence of the present study is indicated with a closed *circle symbol*

**Fig. 7:**
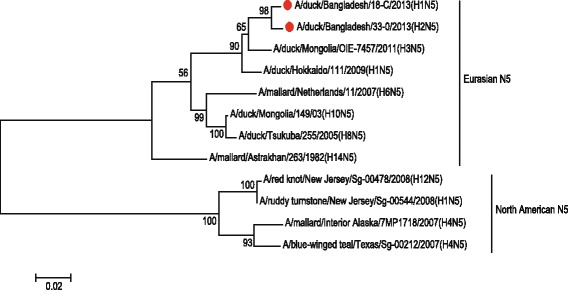
The Maximum Likelihood (ML) phylogenetic tree based on the N5 gene sequences of H1N5 and H2N5 viruses of the present study and representative N5 viruses of different genetic lineages. The *scale* indicates the number of substitutions per site. The H1N5 and H2N5 sequences of the present study are indicated with closed *circle symbols*

The partial H1 gene sequence of the sample 18-C [A/duck/Bangladesh/18-C/2013(H1N5)], H2 gene sequence of the sample 33-O [A/duck/Bangladesh/33-O/2013(H2N5)] and H7 gene sequence of the sample 22-C [(A/duck/Bangladesh/14VIR1121–1/2013(H7N5)], as well as N5 sequences of the sample 18-C and 33-O were subjected to phylogenetic analysis along with downloaded sequences representing different genetic lineages. The phylogenetic tree revealed that the HA gene segments of 18-C (H1N5), 33-O (H2N5) and 22-C or 14VIR1121–1 (H7N5) virus belonged to Eurasian Avian H1 lineage, Eurasian Avian H2 lineage and Eurasian H7 lineage, respectively (Figs. 4, 5 and 6). The H7 sequence of the present strain was different from that of influenza A(H7N9) virus, which caused human infection in China. The NA gene segment of 18-C and 33-O viruses are quite similar and belonged to Eurasian N5 lineage (Fig. 7).

## Discussion

Wild aquatic birds are the natural reservoir of all influenza A viruses [3]. Domestic ducks also have been shown to harbour many subtypes of avian influenza viruses and allow their reassortments [23]. The present study revealed a widespread seroconversion of domestic ducks to Type A influenza virus with an overall seroprevalence of 60.73% in Mymensingh and 47.73% in Sylhet division in 2013. Another study reported 39.76% seroprevalence of type A avian influenza virus in semi-scavenging ducks of Sylhet Hakaluki Haor and Jahangirnagar University Lake areas during winter months of 2009 to 2012 [24]. On further investigation of selected serum samples by HI test for H5 antibodies only 17.5% of ELISA-positive samples were positive for H5 antibodies using HI titre >32 as the cut-off titre for H5-specific antibodies. In West Bengal of India 2.2% ducks were found to be seropositive in HI test for H5 AI considering >40 as the cut-off titre [25].

On virological surveillance only one oropharyngeal swab and five cloacal swabs out of 909 sampled ducks from Mymensingh division were found positive for type A influenza virus M gene by rRT-PCR. No positive sample was found from Sylhet division. This prevalence rate of type A influenza virus in domestic ducks is quite low (0.50%) as compared to that reported by others in domestic ducks of Bangladesh, such as 5.9% [26], 22.05% [24] and 16 out of 39 (41.03%) [27]. This variation might be due to the different time period of sampling. In the present study the samples were collected during January to August, 2013, where as in the previous studies the samples were collected during the period from 2007 to 2012.

All of the 6 M gene positive samples of the present study were negative for H5. Attempted propagation of these M gene positive samples in embryos was not successful with the exception of one sample (22-C). This failure to isolate the virus could be due to very low concentration of virus in the samples. The only embryo-propagated sample 22-C was characterized as low pathogenic H7N5 at OIE-FAO Reference Laboratory, Padova, Italy. Among the remaining 5 M gene-positive but culture-negative swab samples, it was possible to amplify a 640 bp fragment of HA gene and nearly full-length (1413+ bp) NA gene from two samples (18-C and 33-O). The sample 18-C was characterized as subtype H1N5 and the sample 33-O as subtype H2N5. Aquatic birds are known to harbour a wide variety of avian influenza viruses. So the detection of viruses of three different subtypes (H7N5, H1N5 and H2N5) is not surprising. In an earlier study 14 different subtypes of LPAI viruses including H1N1, H1N3, H2N4, H3N2, H3N6, H3N8, H4N2, H4N6, H5N2, H6N1, H6N7, H7N9, H9N2 and H11N3 were identified from domestic ducks of Bangladesh [27]. Isolation of H1N1, H1N2 and H1N3 viruses have been reported from wild and domestic ducks in Korea [28], H1N3 and H1N9 viruses from free-range ducks in Thailand [29] and H2N7 and H2N2 virus from domestic ducks in China [30, 31]. It was interesting that the N gene component of all of the three viruses (H1N5, H2N5 and H7N5) detected in the present study was N5. However, in the previous study [27] all the different N subtypes except N5 were detected in domestic ducks of Bangladesh. Taken together, the findings of these two studies reveal that all the 9 N genes are present in domestic ducks of Bangladesh. Phylogenetically the HA gene of H1N5, H2N5 and H7N5 viruses belonged to Eurasian avian lineages as observed in the previous study [27]. The HA gene of H7N5 was distinct from the HA gene of the newly emerged Influenza A(H7N9) virus. The N5 gene of H1N5 and H2N5 viruses were very similar and also belonged to Eurasian lineage; the NA gene sequence of H7N5 virus was not available.

## Conclusions

Present study shows that 55.12% of the ducks were seropositive for antibodies to Type A influenza virus and a small proportion of Type A influenza virus positive ducks (14 out of 80 randomly selected samples) had previous exposure to H5 subtype viruses, probably H5N1 HPAI or H5N2 LPAI viruses. Active shedding of AI virus at the time of the study was very limited (0.5%). Three viruses were characterized as H1N5, H2N5 and H7N5 subtypes and no H5N1 virus was detected. Findings of the present study warrant further detailed surveillance on avain influenza virus in ducks for better understanding of the ecology of avian influenza viruses in domestic duck population.

## Abbreviations

AI: Avian influenza
ELISA: Enzyme-linked immunosorbent assay
FAO: Food and Agriculture Organization
HA: Haemagglutinin
HI: Haemagglutination inhibition
HPAI: Highly pathogenic
LPAI: Low pathogenic avian influenza
NA: Neuraminidase
OIE: Office international des epizooties
rRT-PCR: Real time Reverse transcription- polymerase chain reaction
RT-PCR: Reverse transcription- polymerase chain reaction
S/N: Sample - negative
WOAH: World Organization for Animal Health
